# A Diagnostic Quandary: Carboplatin-Paclitaxel-Induced Stevens-Johnson Syndrome in a Rare Case of Carcinosarcoma of the Esophagus and Review of the Literature

**DOI:** 10.7759/cureus.47457

**Published:** 2023-10-22

**Authors:** Narendhar Gokulanathan, Pandjatcharam Jagadesan, Chandramouli R, Naadia Nadeem, Sowmya Y Sree

**Affiliations:** 1 Radiation Oncology, Jawaharlal Institute of Postgraduate Medical Education and Research, Puducherry, IND; 2 Radiation Oncology, Krishna Cancer Institute, Cuddalore, IND; 3 Pathology, Jawaharlal Institute of Postgraduate Medical Education and Research, Puducherry, IND; 4 Radiation Oncology, Great Eastern Medical School and Hospital, Srikakulam, IND

**Keywords:** toxic epidermal necrolysis (ten), sarcomatoid carcinoma of head-neck region, aggressive esophageal carcinoma, cervical esophageal cancer, palliative radiotherapy, chemotherapy-related toxicity, chemotherapy protocol carboplatin and paclitaxel with a palliative intent, locally advanced esophageal cancer, stevens-johnson syndrome (sjs)

## Abstract

Sarcomatoid carcinoma of the esophagus, a mixed tumor comprising both carcinomatous and sarcomatoid components and known as carcinosarcoma, is a rare malignancy. Clinically and radiologically, it presents like other esophageal cancers. Here we discuss the case of a 69-year-old male patient with sarcomatoid carcinoma of the esophagus who developed Stevens-Johnson syndrome (SJS) after chemotherapy with carboplatin and paclitaxel. The patient was evaluated for dysphagia and odynophagia. He was initially misdiagnosed to have an esophageal polyp and underwent excision for the same. He presented with recurrent growth at the local site, with histopathological examination showing sarcomatoid carcinoma of the esophagus. After the development of paclitaxel-carboplatin-induced SJS, the patient was subsequently treated with palliative radiotherapy at the primary site for symptomatic relief. He underwent feeding gastrostomy as a supportive nutritional measure and was on best supportive care after a multidisciplinary tumor board discussion. Paclitaxel-carboplatin-induced SJS poses numerous diagnostic conundrums, on account of there being only one reported incident prior to this in literature, to the best of our knowledge. In this report, we explore the diagnostic and therapeutic predicaments associated with a rare disease that is under-reported and understudied in literature and delve into the various treatment modalities that can benefit the patients. The case also demonstrates the delicate balance between cancer chemotherapeutics and their Pandora’s box of adverse effects.

## Introduction

Sarcomatoid carcinoma of the esophagus is a rare disease with an incidence of <2% among all esophageal cancers. It is clinically and radiologically consistent with squamous and adenocarcinomas of the esophagus, making it difficult to diagnose a particularly aggressive disease without a tissue diagnosis [1]. It presents as a polypoidal mass with dysphagia and loss of weight. Histopathological examination shows components indicative of both carcinomatous and sarcomatoid pathology [2]. Surgery is the primary modality of treatment. Chemotherapy or radiation can be used as adjuvant modalities to enhance disease control. Taxane and antimetabolite-based chemotherapy are commonly used for this malignancy [3].

This report describes the management of a patient with sarcomatoid carcinoma of the esophagus and also describes the development of paclitaxel-carboplatin-induced Stevens-Johnson syndrome (SJS) in this patient and the diagnostic conundrums it poses, on account of there being only one such reported incident, to the best of our knowledge, prior to this in literature [4]. The case highlights the delicate balance between cancer chemotherapeutics and their numerous adverse effects.

## Case presentation

A 69-year-old male patient with no known comorbidities presented with progressive dysphagia to solids and associated odynophagia over two months. He had a swelling in the midline of the neck, which gradually increased in size.

Video laryngoscopy revealed a polypoidal mass in the left pyriform fossa. Contrast-enhanced computed tomography (CECT) showed a polypoidal growth of 5.4x2.5x3 cm in the anterolateral wall of the upper esophagus at the C5-T1 vertebral level protruding into the lumen. The lesion had a stalk-like heterogeneous enhancement from the anterior wall to the central portion of the lesion. The trachea was pushed away from the midline by the mass. There was involvement of locoregional lymph nodes. Upper gastrointestinal (UGI) endoscopy showed a mobile polypoidal growth of diameter 5cm on the right side of the cervical esophagus.

A biopsy of the lesion revealed severe squamous dysplasia. The patient underwent pharyngotomy by transcervical approach and complete polyp excision at another hospital before being reviewed at our institute. The excised specimen was a friable polyp measuring 6x6 cm. The patient was referred to our institute for further evaluation one week after this.

**Figure 1:**
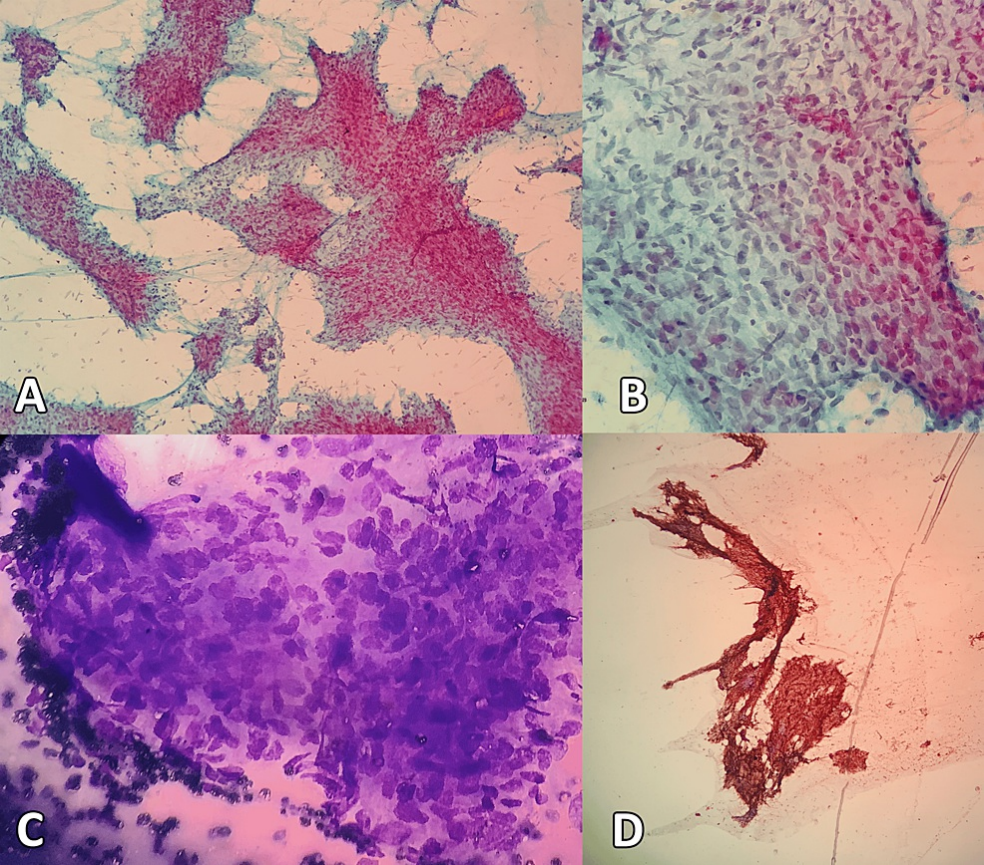
(A) Fine needle aspirate smears were cellular and showed tissue fragments and cohesive clusters of tumor cells (Papanicolaou stain, 100×); (B) Tumor cells were moderately pleomorphic and prominently spindled in morphology with coarse chromatin and bipolar cytoplasm (Papanicolaou stain, 400×); (C) Tumor cells were moderately pleomorphic and prominently spindled in morphology with coarse chromatin and bipolar cytoplasm (May Grunwald-Giemsa stain, 400×); (D) Immunocytochemistry for pancytokeratin was strongly positive in the tumor cells (Diaminobenzidine stain, 100×).

Histopathological examination revealed a polypoidal specimen with surface ulceration and necrosis, measuring 4.2x1.5 cm. Microscopic examination (Figure 1) showed tumor cells arranged in sheets of spindle to oval cells exhibiting severe pleomorphism, with hyperchromatic nuclei, increased mitosis (5-6/high power fields) with necrosis, and interspersed keratin pearls. Immunohistochemistry showed immunoreactivity to cytokeratin (2+) and vimentin (4+), and a diagnosis of carcinosarcoma was made. The margin of the specimen was free of tumor. Lymph nodal dissection was not done as it was initially assumed to be a polyp.

After discussion with the multidisciplinary tumor board, the patient was planned for adjuvant paclitaxel and carboplatin chemotherapy. Prechemotherapy blood investigations were within normal limits, and echocardiography showed normal ventricular function. One month after the surgery, he received the first cycle of paclitaxel (280 mg) with carboplatin (420 mg) with appropriate premedication consisting of dexamethasone, diphenhydramine, ranitidine, and ondansetron over a non-DEHP (di(2-ethylhexyl) phthalate) tubing, using a 0.15-micron filter. He tolerated chemotherapy well, and there was no hypersensitivity reaction or anaphylaxis.

**Figure 2:**
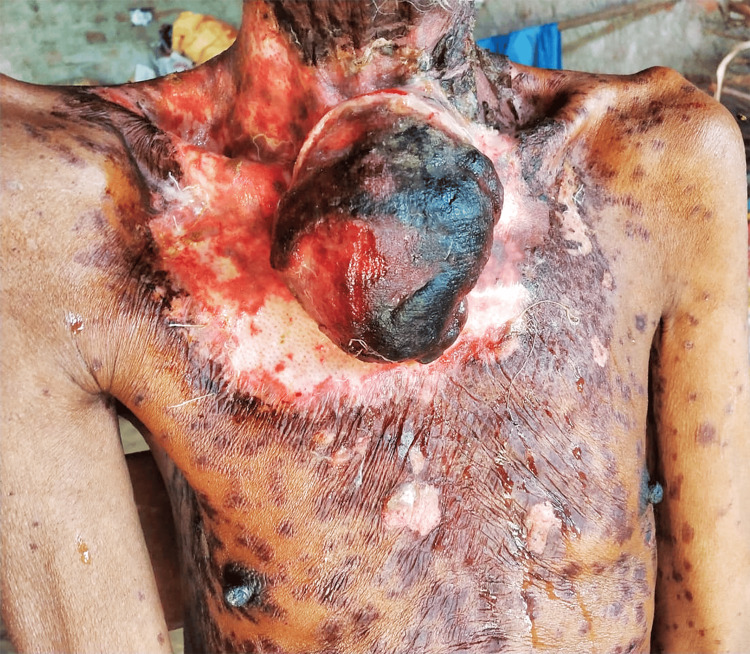
Sarcomatoid carcinoma of esophagus breaching skin anteriorly, surrounded by healing bullous lesions and exfoliated skin.

Two weeks after the first cycle of chemotherapy, the patient presented with a rapid increase in the size of the disease. The tumor grew anteriorly and breached the skin, resulting in external bleeding. He developed oral mucositis, multiple painful erythematous lesions mixed with partially confluent bullous lesions over the trunk and extremities, with exfoliation, skin peeling, and fever (Figure 2). Due to the development of intolerance to chemotherapy, further chemotherapy was deferred keeping in mind the advanced age of the patient.

Hemostatic radiation of 8Gy/1# to the primary site was planned. The bleeding stopped after the first fraction of radiotherapy was delivered, and the patient was admitted for evaluation and management of the skin lesions. One more fraction of 8Gy/1# was delivered to the primary site after a one-week interval. His hemogram and electrolytes were within normal limits, and tests returned negative for cold agglutinins and eosinophilia. He did not have leucocytosis or elevation of liver enzymes, ruling out drug reaction with eosinophilia and systemic symptoms (DRESS) syndrome.

The patient was started on intravenous fluids and barrier nursing, with supportive care and regular dressing of the wounds with antiseptic. Nikolsky’s sign and Asboe-Hensen bulla spread signs were positive. Ophthalmologic evaluation revealed a pseudomembrane with discharge and congestion in the upper palpebral conjunctiva with matting of lashes (Grade 3 conjunctivitis). Subsequent management of the same was done with adequate analgesic cover using morphine.

A biopsy was taken from the bullous skin lesions, and it showed epidermal necrosis with occasional apoptotic keratinocytes. Superficial dermis showed moderate lymphocytic infiltrates admixed with few eosinophils in the periadnexal and perivascular region, with extravasation of red blood cells. Immunofluorescence, IgG, IgA, and C3, were negative in the intraepidermal and dermoepidermal junction. The histopathological features correlated with the clinical picture and suggested SJS.

The patient was also worked up for other likely causes of skin lesions. He did not fit the European Registry of Severe Cutaneous Adverse Reactions to Drugs and Collection of Biological Samples (RegiSCAR) criteria for diagnosing DRESS. He had not received beta-lactams previously, effectively ruling out acute exanthematous pustulosis. A biopsy along with immunofluorescence testing ruled out paraneoplastic pemphigus, linear IgA disease and eccrine squamous syringometaplasia. Chikungunya was ruled out using enzyme-linked immunoassay (ELISA) serological testing. Toxic erythema of chemotherapy, caused by cytokine-driven keratinocyte damage, was ruled out clinically based on the truncal location of the lesions, lack of neuropathic or dysesthesia component and the lack of exposure to antimetabolite chemotherapy [5].

The patient was on the following drugs: paracetamol, famotidine, vitamin B complex, and mucaine gel. He was not on anti-epileptics, sulpha-drugs, antivirals, antiretrovirals, antifungals, or non-paracetamol non-steroidal anti-inflammatory drugs (NSAIDs). The algorithm of drug causality for epidermal necrolysis (ALDEN) scoring was used as a surrogate for diagnosing SJS, and the score was 3 (Table 1), suggesting a possible drug causality [6].

**Table 1:** ALDEN scoring for causality of Stevens-Johnson syndrome. ALDEN: algorithm of drug causality for epidermal necrolysis

Criterion	Definition	Score
Delay from initial drug component intake to onset of reaction (index day)	5-28 days	+3
Drug present in the body on Index day	Drug is stopped at a time point prior to the index day by more than five times the elimination half-life but liver or kidney function alterations or suspected drug interactions are present	-1
Prechallenge/rechallenge	No previous exposure to drug	0
Dechallenge	Drug is stopped	0
Type of Drug	Suspected association with several previous reports of adverse effects available	+1

**Figure 3:**
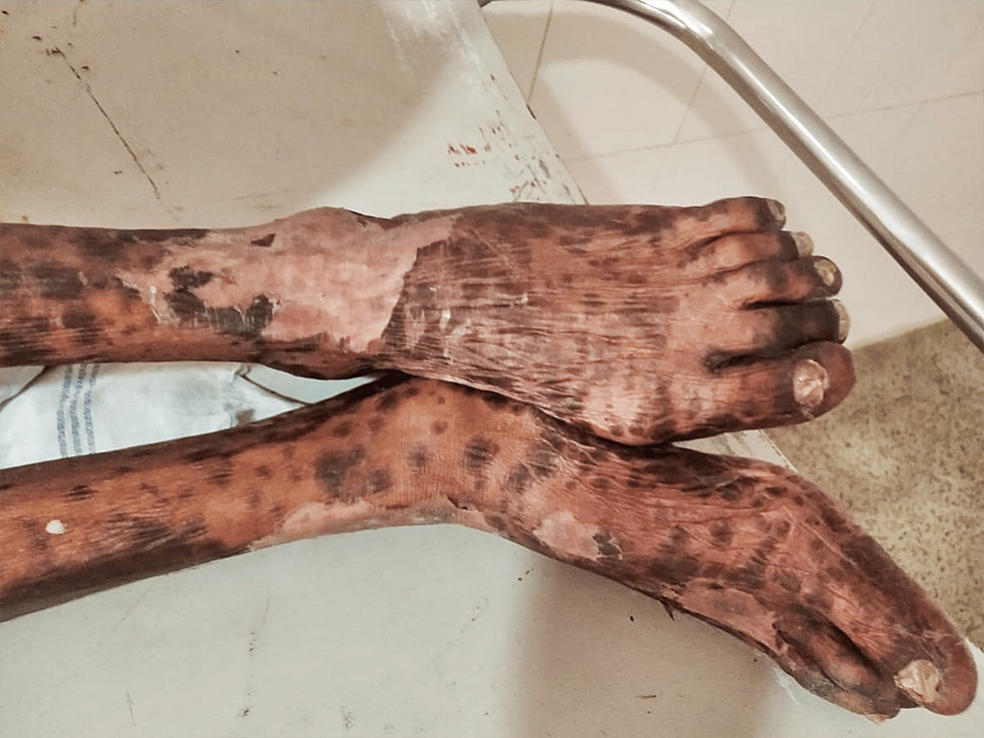
Healing Stevens-Johnson syndrome lesions.

Due to the onset of SJS, the patient could not regain fitness for the second cycle of chemotherapy. A week later, he received one more fraction of 8Gy to the gross tumor, and there was no subsequent bleeding. There was no subsequent reduction in the size of the primary tumor in the following week, and the patient continued to receive regular dressing and barrier nursing (Figure 3).

**Figure 4:**
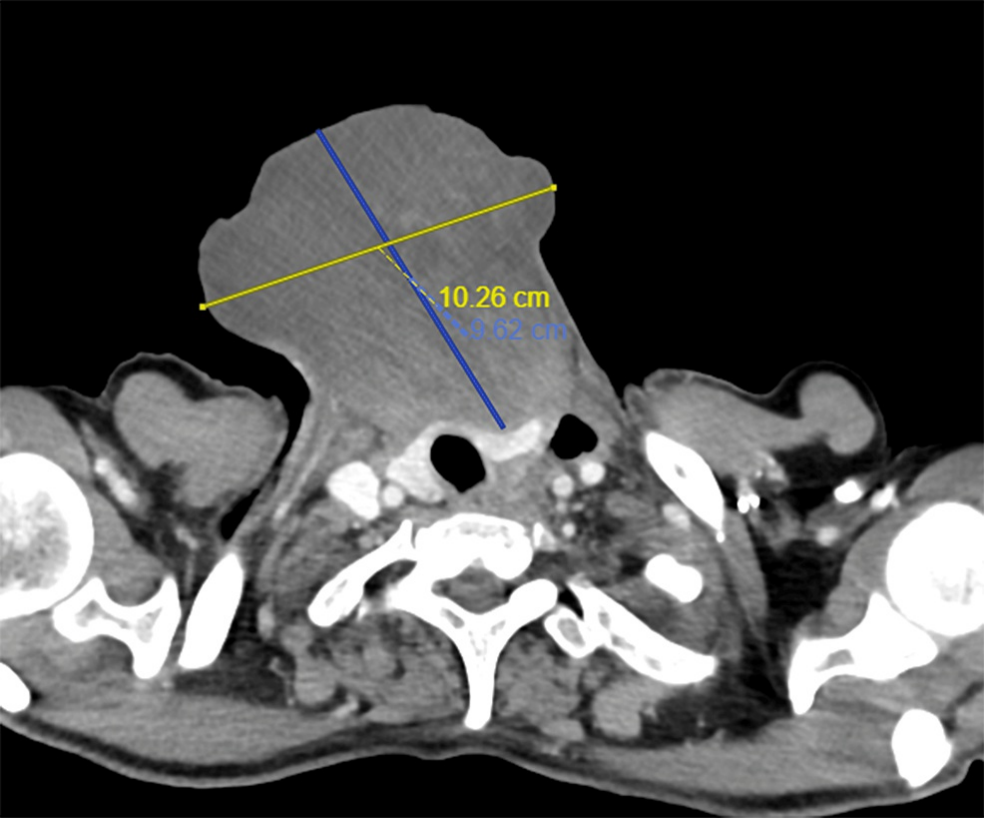
An axial section of the sarcomatoid carcinoma of esophagus, as seen on CECT imaging. CECT: contrast-enhanced computed tomography

A CECT was taken, which showed a well-defined heterogeneously enhancing soft tissue dense lesion in the anterior aspect of the neck measuring 9.3x9.7x10.2 cm (anteroposterior x mediolateral x craniocaudal) (Figure 4). The lesion had indistinct fat planes with strap muscles and the left lobe of the thyroid and caused an indentation on its anterior aspect. The cervical esophagus was displaced anterolaterally on the left side, anterior to the common carotid artery, and lateral to the left lobe of the thyroid, which had indistinct fat planes with esophagus, possibly due to traction by the lesion. The tumor did not seem to have responded to the chemotherapy or radiotherapy. It had increased in size, suggesting a progressive disease. However, there were no subsequent bleeding episodes.

Following the resolution of mucositis and the bullous skin lesions, two weeks later, a plan for feeding gastrostomy and nutritional supplementation was discussed in the multidisciplinary tumor board. As the patient’s performance decreased, he and his family were counseled regarding palliative and best supportive care. The patient survived for four months after this.

## Discussion

Carcinosarcoma or sarcomatoid carcinoma of the esophagus, described first by Virchow in 1865, is a rare entity composed of epithelial and mesenchymal components. Previous studies have reported variable prognosis and survival, with attention to its low rate of nodal spread and invasiveness and propensity for hematogenous spread. It comprises only 0.26-2% of all esophageal cancers [1].

It is more common in men, with a predilection towards alcohol and tobacco users. It usually presents as a pedunculated polypoidal, fleshy mass causing dysphagia and odynophagia, progressing to obstruction, anorexia, and loss of weight [1]. Sessile and broad-based lesions show wide invasion. It usually presents in the mid and distal esophagus, with proximal esophagus involvement, as in our patient, accounting for <10% of carcinosarcoma cases. Lymph nodal involvement offers an additional point in favor of diagnosing sarcomatoid carcinoma over sarcoma [3,7].

Microscopically, it has a biphasic appearance with squamous epithelial components and spindle elements, with the latter arranged in a fascicular pattern. Both the histological variants may be present in varying proportions, and adequate tissue is required to arrive at an accurate diagnosis. In the absence of distinct histological features, immunohistochemistry markers are utilized for diagnosis, which includes cytokeratin, p63, smooth muscle actin, and vimentin, with comparable Ki-67 labeling indices around 35-40% [2]. Positivity for markers ranges from focal or patchy to strong. Care should be taken to differentiate sarcomas from sarcomatoid carcinomas.

There is insufficient data to arrive at the appropriate treatment modality. Present paradigms keep surgery at the forefront and have been designed to keep carcinosarcoma of the uterus as a model. It is mandatory to provide nutritional support prior to any treatment offered to the patient. Oral supplementation is preferred, and nasogastric or nasojejunal tube feeding is considered a second-line measure. As with conventional non-metastatic esophagus tumors, a trimodal approach of neoadjuvant chemoradiotherapy with a radiation dose of 40-50 Gy to reduce tumor volume, followed by R0 esophagectomy with regional lymphadenectomy as required would be the treatment of choice. In patients deemed unfit for surgery, definitive radiotherapy is recommended with a dose of 50-70 Gy. If unfit for definitive radiotherapy, palliative chemotherapy and radiotherapy could be used as alternatives. Esophageal stenting can be done to relieve dysphagia and improve quality of life. Common sites of distant metastases include lungs, pleura, liver, brain, and bone, with greater tumor size at diagnosis being a poor prognostic factor [3,7,8].

The five-year cancer-specific survival rate of patients treated with radical surgery ranged from 44.8% to 61.9% [9]. Despite a higher three-year survival rate and curative resection rate, the five-year survival rate of carcinosarcoma was comparable to squamous cell carcinoma (26.7% vs 22.4%). Adjuvant fluorouracil-based chemotherapy and chemoradiotherapy are recommended in lymph node-positive patients, keeping node-negative patients on regular monitoring. Recent trials have suggested the use of immunotherapy like pembrolizumab, camrelizumab, and nivolumab in concurrence with chemotherapy or second-line agents [8,10].

Among anticancer drugs causing SJS, paclitaxel has been a documented cause only once, in 2004 [4], and there have been no documented cases of carboplatin-induced SJS in the literature. The mechanism by which paclitaxel, an antimicrotubular agent, could cause SJS is unclear. Carboplatin-induced platinum-DNA complexes could be immunogenic and cause SJS. Like antimetabolites, it could be attributed to a direct toxicity effect of the drug, but no evidence has been described in the literature so far. Incidences of combination therapy of paclitaxel and carboplatin causing SJS have been documented in the literature, albeit associated with other confounding drugs like pemetrexed and penicillin. Through the clinical profile of the patient, ALDEN score, a temporal association, and a panel of tests to rule out differentials, we have attributed this SJS to a combination of carboplatin-paclitaxel. There have been case reports of SJS associated with docetaxel reported in the Food and Drug Administration Adverse Event Reporting System (FAERS) database [11-13].

The adverse effects usually attributed to paclitaxel are alopecia, onycholysis, pancytopenia, hypersensitivity reactions, neuropathic manifestations, cardiovascular events, and enteritis-like features, while carboplatin causes myelosuppression, GI features, and electrolyte imbalance [14-16]. Numerous chemotherapy manuals and pharmacological textbooks have not mentioned SJS as a complication of paclitaxel or carboplatin chemotherapy. SJS, an immune-driven condition, has life-threatening implications and widespread involvement of skin and mucosa and could deter oral feeding in an already nutrition-compromised, cachexic patient with esophageal cancer. It requires immediate and comprehensive supportive care, daily wound management with antiseptics and sterile precautions.

Since SJS is a hitherto unheard-of yet grave adverse effect of paclitaxel-carboplatin combination chemotherapy, it must be on every oncologist’s differential diagnosis to ensure an early call to action and management. The patients at risk might need to undergo human leukocyte antigen (HLA) testing and the utility of alternative chemotherapeutics.

## Conclusions

For a rare cancer variant where the treatment paradigm has not been established yet, a multidisciplinary tumor board discussion is mandatory to ensure appropriate management. The patient’s performance status and treatment tolerability should be factored into making clinical decisions for disease management. Nutritional support acquires prime importance, especially in patients with esophageal carcinoma. Care should be taken to ensure that the patient can tolerate changes due to cytotoxic anti-cancer treatment. As cancer evolves, so should the treatment paradigm; early incorporation of palliative care also becomes necessary for good patient outcomes.

We must constantly watch for life-threatening complications and adverse effects of the cytotoxic chemotherapy regimens to identify them as early as possible to offer appropriate treatment at the right time to ensure patient safety.

## References

[1] Au JT, Sugiyama G, Wang H, Nicastri A, Lee D, Ko W, Tak V (2010). Carcinosarcoma of the oesophagus - a rare mixed type of tumor. J Surg Case Rep.

[2] Sano A, Sakurai S, Kato H (2009). Clinicopathological and immunohistochemical characteristics of esophageal carcinosarcoma. Anticancer Res.

[3] Guy CD, Cardona DM, Pittman PD, McCall SJ, Zhang X (2016). Sarcomatoid carcinoma of the esophagus. JSM Gastroenterol Hepatol.

[4] Hiraki A, Aoe K, Murakami T, Maeda T, Eda R, Takeyama H (2004). Stevens-Johnson syndrome induced by paclitaxel in a patient with squamous cell carcinoma of the lung: a case report. Anticancer Res.

[5] Bolognia JL, Cooper DL, Glusac EJ (2008). Toxic erythema of chemotherapy: a useful clinical term. J Am Acad Dermatol.

[6] Sassolas B, Haddad C, Mockenhaupt M (2010). ALDEN, an algorithm for assessment of drug causality in Stevens-Johnson Syndrome and toxic epidermal necrolysis: comparison with case-control analysis. Clin Pharmacol Ther.

[7] Tustumi F, Uema RH, Takeda FR (2017). Esophageal carcinosarcoma: single institute experience and review of the literature of this rare entity. Acta Gastroenterol Latinoam.

[8] Gong H, Li B (2021). Guidelines for radiotherapy of esophageal carcinoma. Precis Radiat Oncol.

[9] Li P, Li Y, Zhang C (2021). Clinicopathological and prognostic characteristics of esophageal spindle cell squamous cell carcinoma: an analysis of 43 patients in a single center. Front Oncol.

[10] Iyomasa S, Kato H, Tachimori Y, Watanabe H, Yamaguchi H, Itabashi M (1990). Carcinosarcoma of the esophagus: a twenty-case study. Jpn J Clin Oncol.

[11] Rosen AC, Balagula Y, Raisch DW (2014). Life-threatening dermatologic adverse events in oncology. Anticancer Drugs.

[12] Diab O, Mcentire D, Kassim T (2019). Docetaxel-induced Stevens-Johnson syndrome in a patient with metastatic prostate adenocarcinoma. Case Rep Oncol Med.

[13] (2023). CTEP: Possible side effects of carboplatin and paclitaxel. https://ctep.cancer.gov/protocolDevelopment/docs/regimes/SideEffects-Carboplatin-Paclitaxel.docx.

[14] Sibaud V, Lebœuf NR, Roche H (2016). Dermatological adverse events with taxane chemotherapy. Eur J Dermatol.

[15] (2023). Paclitaxel - Drug Monograph, BCCA. Paclitaxel - Drug Monograph.

[16] (2023). Carboplatin - Drug Monograph, BCCA. Carboplatin - Drug Monograph.

